# Enhanced Conductivity and Antibacterial Behavior of Cotton via the Electroless Deposition of Silver

**DOI:** 10.3390/molecules26164731

**Published:** 2021-08-05

**Authors:** Changkun Liu, Dan Liao, Fuqing Ma, Zenan Huang, Ji’an Liu, Ibrahim M. A. Mohamed

**Affiliations:** 1 College of Chemistry and Environmental Engineering, Shenzhen University, Shenzhen 518060, China; 2060222001@email.szu.edu.cn (D.L.); 2172226014@email.szu.edu.cn (F.M.); 2172226010@email.szu.edu.cn (Z.H.); 2011140021@email.szu.edu.cn (J.L.); 2 Shenzhen Key Laboratory of Environmental Chemistry and Ecological Remediation, Shenzhen University, Shenzhen 518060, China; 3 Department of Chemistry, Faculty of Science, Sohag University, Sohag 82524, Egypt; ibrahim2008@szu.edu.cn

**Keywords:** cotton, electroless deposition, SI-ATRP, conductive property, antibacterial property

## Abstract

In this study, the surface-initiated atom transfer radical polymerization (SI-ATRP) technique and electroless deposition of silver (Ag) were used to prepare a novel multi-functional cotton (Cotton-Ag), possessing both conductive and antibacterial behaviors. It was found that the optimal electroless deposition time was 20 min for a weight gain of 40.4%. The physical and chemical properties of Cotton-Ag were investigated. It was found that Cotton-Ag was conductive and showed much lower electrical resistance, compared to the pristine cotton. The antibacterial properties of Cotton-Ag were also explored, and high antibacterial activity against both *Escherichia coli* and *Staphylococcus aureus* was observed.

## 1. Introduction

Natural cellulose materials are used in a wide range of applications due to their low cost [1,2,3]. The modification of cellulose to obtain new functionalities has been a focus of both academic and industrial research. Modified cellulose is used for different applications, including electromagnetic shielding [4,5], UV-protection [6], antifouling media [7], oil–water separation [8], antibacterial media [9,10], photocatalysis [11], supercapacitors [12] lithium–sulfur batteries [13] and flexible sensors [14].

As a natural cellulose material, the cotton possesses the advantages of low cost, large production volume and ease of surface modification. Highly conformal nanoscale metal coatings on cotton have been shown to improve the performance of cotton-based electronics [15]. A variety of methods can be used to increase the electrical conductivity of cotton, including in situ polymerization [16], spray assembly [17], metal plating [18], and dip coating [19]. In addition to these methods, electroless deposition (ELD) has high potential for the coating of metals on cotton fabrics, due to its low cost, ease of design and convenience for use on an industrial scale. ELD can be used to deposit different metals on various substrates, such as plastic, paper and metal oxides [18,20,21].

With the rapid industrial development and the improvement of living standards, antibacterial performance has attracted increasing attention. People are inevitably exposed to a variety of bacteria, fungi and other microorganisms. These microorganisms may rapidly grow and multiply under appropriate environmental conditions, spread diseases through contact and affect human health. In daily life, the cotton is often a suitable location for the survival of these microbes and an important source of disease transmission. Therefore, investigation of the antibacterial performance of cotton is of great significance.

The combination of conductivity and the antibacterial effect in a single multi-functional material has great potential for use in industrial, civilian, military and other fields [21,22]. In particular, fabrics possessing both conductive and antibacterial properties can be used in smart wearable equipment. For example, woven fabrics can be inserted as the interlayer inside the smart wearable equipment, conducting electricity for cooling, heating or other purposes, while also functioning the antibacterial ability for sustainable use. The combination of silver and cotton-based materials has been widely reported. For example, Ma et al. [23] reported the silver-plated cotton/spandex blended fabric material for wearable antibacterial strain sensors. Liu et al. [24] fabricated silver-plated cotton fabrics (SPCFs) through polydopamine reduction and glucose reduction reaction for wearable antibacterial and intelligent textiles. Wang et al. [25] reported a conductive cotton fabric with excellent washability, as fabricated by electroless deposition of silver on the surface of fibers after silane modification. Tan et al. [26] prepared Ag@CFs non-woven fabrics for wearable electromagnetic shielding material. Therefore, silver-coated cotton possesses both electrical conductivity and antibacterial properties. However, no research work has been published for the preparation of silver-coated cotton materials via the method of polymer brush grafting and the subsequent electroless deposition of silver. The polymer brush grafting step before the electroless deposition of silver would provide more deposition sites for silver from the pendent groups of the grafted polymer brushes, in an effort to effectively enhance the silver deposition efficiency. In addition, the electroless deposition technique via the chemical method is easy to operate, without the need of electricity.

In this work, a silver functionalized cotton material (Cotton-Ag) was successfully prepared, using a combination of polymer brush grafting via the surface-initiated atom transfer radical polymerization (SI-ATRP) technique and electroless deposition. The poly(methylpropenyloxyethyl trimethylammonium chloride) (pMETAC) was grafted onto cotton surface via SI-ATRP, which provided more grafting sites for silver deposition. The surface morphology and the thermal, conductive and antibacterial properties of Cotton-Ag were investigated. This research provides insight into the preparation of multi-functional cotton fabrics for potential applications in the field of smart wearable equipment.

## 2. Experimental

### 2.1. Materials

Cotton was purchased from Tianhong Sanitary Material Co., Ltd. (Anqing, China). Dichloromethane (DCM, AR) was purchased from Baishi Chemical Co., Ltd. (Tianjin, China). 2-Bromoisobutyryl bromide (BIBB, 98%), pyridine (≥99%), methacryloxyethyl trimethyl ammonium chloride (METAC, 75 wt%), 2,2-bipyridine (99%), cuprous bromide (CuBr, 99%), copper bromide (CuBr_2_, 99%), ammonium chloropalladite (Pd ≥ 36.5%), sodium hydroxide (NaOH, AR), cupric sulfate (AR, 99%), potassium sodium tartrate (AR, 99%), silver nitrate (AR, 99.8%) and Tween-80 (cell culture grade) were purchased from Aladdin (Shanghai, China). The biochemical beef extract, tryptone and technical agar powder reagents were obtained from Huankai Microbial Technology Co., Ltd. (Guangdong, China). All of the reagents used in this study were of analytical grade. Deionized (DI) Water was produced in an ultrapure water system (UPR-II-10T, Ulupure Technology Co., Ltd., Chengdu, China) and was used in all experiments performed in this study.

### 2.2. Material Preparation

#### 2.2.1. Preparation of the Cotton-Br 

**Figure 1:**
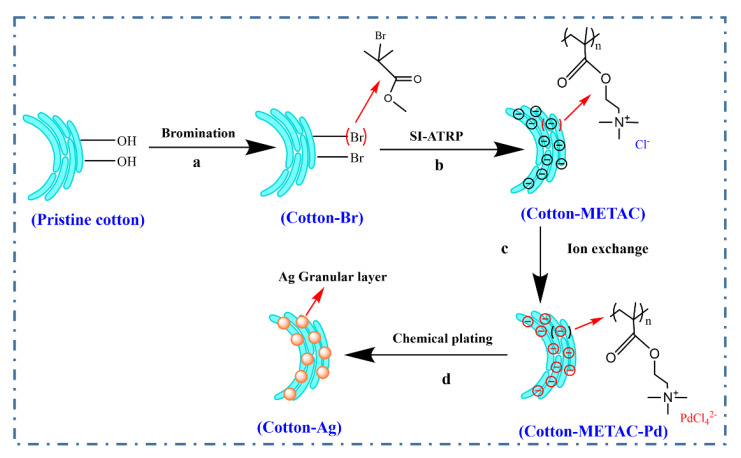
Schematic diagram of the preparation process of (**a**) bromination of pristine cotton, (**b**) methacryloxyethyltrimethyl ammonium chloride modification of Cotton-Br, (**c**) preparation of Cotton-METAC-Pd, and (**d**) Ag-plating of Cotton-METAC-Pd for the preparation of Cotton-Ag.

The cotton sample (0.3 g) was weighted with an electronic balance and placed in a 50 mL beaker. Then, the cotton was cleaned with 10 mL of DCM to remove the excess pollutants on its surface. After that, the cotton was removed from the beaker and squeezed to remove the excess liquid. Next, the cotton was placed in a 50 mL centrifuge tube, and 15 mL of DCM was added. In a fume hood, the centrifuge tube was placed in an ice-water bath in a crystalizing dish, and then 3 mL of BIBB and 1 mL pyridine were slowly added and mixed with gentle stirring, using a clean glass rod. After that, the mixture was sealed and stirred under magnetic stirring (SP-18, Hangzhou Mio Instrument Co., Ltd., Hangzhou, China) for 1 d at room temperature with a rotation speed of 300 r/min. After the reaction was completed, the cotton was removed and washed with deionized water 3–4 times, and then soaked with acetone solution until no yellow organic solution was present on the cotton. Finally, the modified cotton was blown dry with argon to obtain cotton-Br (Figure 1a).

#### 2.2.2. Preparation of Cotton-METAC 

Cotton-METAC was prepared according to a previous method [27] with slight modification. Cotton-Br (0.2 g) was weighed into a clean tube together with a magnetic stirrer, and the tube was aerated with argon for 5 min. Subsequently, methanol (5 mL) and METAC (4.6 mL) were successively added to the test tube, and the silicon stopper was plugged. Then, the system was homogenized with slight stirring, and 2,2-dipyridyl (0.48 g), CuBr (0.12 g), and CuBr_2_ (0.019 g) were added to the system. The tube was covered with a silicon stopper and the mixture was stirred evenly again. Then, the test tube was fixed on a thermostatically-heated magnetic stirrer, and the reaction was carried out in a water bath at 60 °C at a rotation speed of 300 r/min for 10 h. After the reaction was completed, the cotton was removed, and washed with methanol until it became white, then washed with water three times, and finally dried with argon gas to obtain cotton-METAC (Figure 1b).

#### 2.2.3. Preparation of Cotton-METAC-Pd

Cotton-METAC (0.1 g) was weighed into 5 mM (NH_4_)_2_PdCl_4_ aqueous solution (25 mL), stirred for 30 min and then removed, and the cotton was washed 4–5 times with deionized water to remove the free Pd^2+^ from the cotton. In the reaction process, PdCl_4_^2−^ was adsorbed on the surface of the material through ion exchange and the intermediate, and Cotton-METAC-Pd product was obtained (Figure 1c). The Pd on the surface can be used as a catalyst for silver electroless deposition.

#### 2.2.4. Preparation of Copper Electroless Deposition Solution

Two aqueous solutions labeled A and B were used. The A solution is a liquid containing 12 g/L sodium hydroxide NaOH, 13 g/L copper sulfate pentahydrate (CuSO_4_·5H_2_O), and 29 g/L potassium sodium tartrate (KNaC_4_H_4_O_6_·4H_2_O). The B solution is a 0.95% formaldehyde aqueous solution. The aqueous solutions of A and B were mixed in a volume ratio of 1:1 and were used as the copper electroless deposition solution at room temperature [28].

#### 2.2.5. Preparation of Silver Electroless Deposition Solution

There were two aqueous solutions: C and D. The C solution is a 2 g/L silver ammonia solution, and was obtained as follows. AgNO_3_ (0.2 g) was weighed and then dissolved in deionized water (100 mL), and then 2 drops of 10% NaOH were added to the solution. The solution showed a brown-yellow precipitate, and ammonia was continuously added to the solution until the precipitation disappeared to obtain the 2 g/L silver ammonia solution. The D solution is a 5 g/L sodium potassium tartrate solution. The aqueous solutions C and D were mixed in a volume ratio of 1:1 and were used as a silver electroless depostion solution at room temperature [28].

#### 2.2.6. Preparation of the Cotton-Ag

Cotton-METAC-Pd (0.1 g) was placed in freshly prepared copper electroless deposition solution (50 mL) for 30 min until the solution color became brown-red, and the cotton was removed and soaked in a freshly prepared silver electroless deposition solution (50 mL). At the end of the reaction, the transparent silver electroless deposition solution turned into a blue solution, and the loaded copper cotton changed from brownish red to gray (Figure 1d).

### 2.3. Material Characterization

The prepared cottons with the loaded metal were washed several times to ensure that the metal salt on the surface of the cotton was removed. Then, the surface morphologies of the cottons were observed, using a scanning electron microscope (SEM, S-3400N(II), JEOL, Tokyo, Japan). The functional groups of the pristine cotton and Cotton-METAC were identified by Fourier transform infrared spectroscopy (FTIR, IR Affinity-1, Shimadzu Corporation, Kyoto, Japan). The surface element chemical information of the cotton at each stage were characterized by X-ray photoelectron spectroscopy (XPS, ESCALAB 250XI, Thermo, Waltham, MA, USA). The thermal stability of the modified-cotton fiber was tested, using a STA409PC synchronous thermal analyzer (TG-DTA, Selb, Germany) at the following test conditions: heating rate of 10 °C/min, nitrogen atmosphere, and temperature range of 30–1000 °C. The weight of the used cotton fiber sample was 0.2 g, and a pellet with a diameter of 2 cm was formed by pressing with a tableting machine under a pressure of 5 MPa for 30 s. The resistance of the pellet was measured with a digital multimeter (15B, Shanghai Shilu Instrument Co., Ltd., Shanghai, China) with the unit of Ω. Each group of samples was measured 10 times and the average values were reported.

The weight of the cotton before and after the metal loading was measured using a Sartorius precision electronic balance. The mass of the cotton fiber before loading was recorded as $W_{1}$, and the mass of the cotton after loading was recorded as $W_{2}$. The weight gain rate of the cotton after loading the metal is given by the following:

(1) $$W=\frac{W_{2}-W_{1}}{W_{1}}\times 100\%$$

### 2.4. Antibacterial Behavior

Gram-negative bacteria (*Escherichia coli*) and Gram-positive bacteria (*Staphylococcus aureus*) were selected as the experimental strains and were provided by the Microbiology Basic Teaching Laboratory of the School of Chemistry and Environmental Engineering of Shenzhen University ((1) *E. coli*: Gram-negative Brevibacterium, size 0.5 × 1–3 μm. (2) *S. aureus*: Gram-positive stain. Spherical, diameter of approximately 0.8 μm).

## 3. Results and Discussion

**Figure 2:**
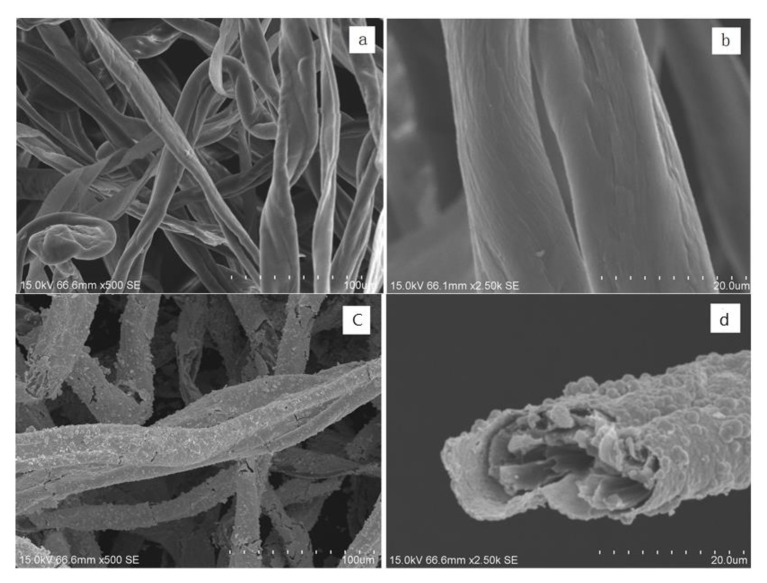
SEM images of (**a**,**b**) pristine cotton and (**c**,**d**) modified cotton.

In this work, the modified cotton was prepared with conductivity and antibacterial property as shown in Figure 1. The morphology of the cotton before and after modification was investigated with the results shown in Figure 2. The surface of the original cotton is smooth, and at the same time, some cotton may have a flat ribbon shape with a spiral-like twist, which is the natural form of cotton during the process of growth, known as the “natural transformation”. Natural twists are a unique morphological feature of the cotton that can be used to distinguish cotton from other fibers. After silver electroless deposition, the surface of the cotton is covered with a continuous and dense silver coating. As can be more clearly seen in Figure 2d, the cotton’s surface is tightly deposited, and the particles are even and there are no traces of peeling. This indicates the presence of a continuous layer of metal on the surface of the cotton. Therefore, the morphology of the modified cotton verifies the successful formation of Cotton-Ag.

**Figure 3:**
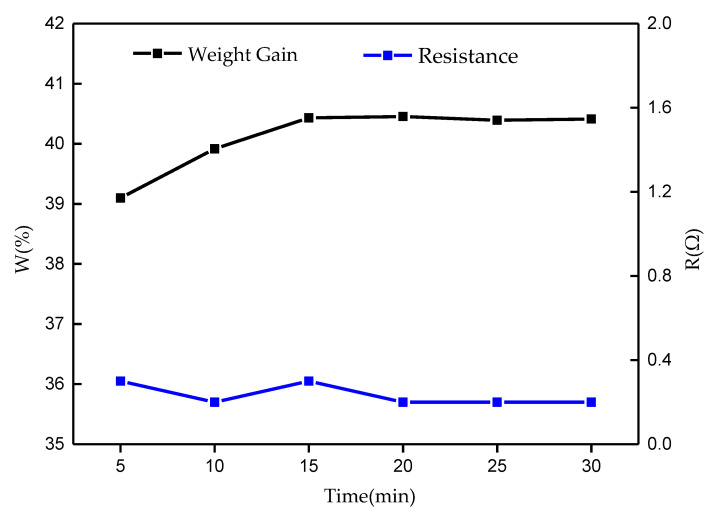
Effect of Ag-deposition time on the resistance and weight gain of the modified cotton.

The effect of the deposition time was studied with the results displayed in Figure 3. The weight gain rate shows an initial increase during 5–15 min and remains stable after 15 min. The surface resistance of the cotton also fluctuates in a small range, and remains stable after 20 min. The results indicate that the surface of the modified cotton is fully covered by silver and gains a stable conductive performance at the deposition time of 20 min, which is regarded as the optimal deposition time for the modified cotton in this study. 

**Figure 4:**
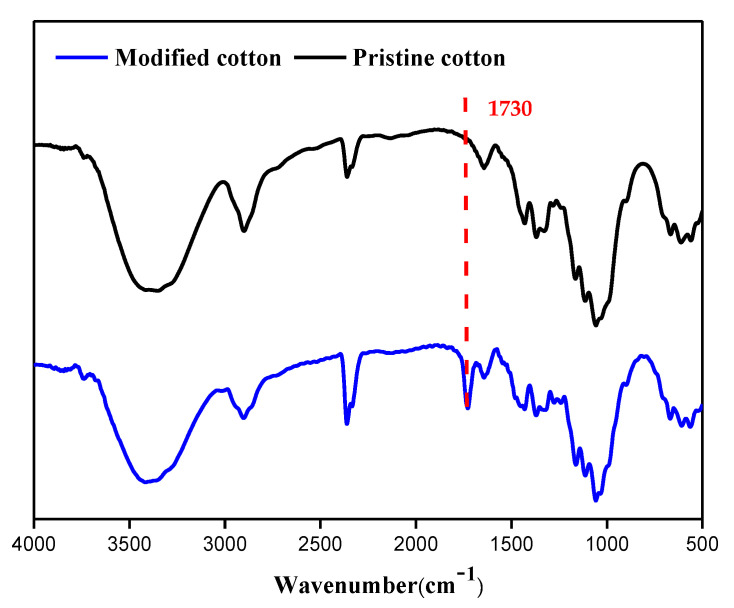
FT-IR spectra of the cotton before and after modification.

FTIR is a useful technique for the study of the functional groups of materials. For the infrared spectrum of the pristine cotton presented in Figure 4, a relatively broad absorption peak around 3350 cm^−1^ is the characteristic peak of OH on the cotton surface and the absorption peaks at 1161 cm^−1^ and 1109 cm^−1^ are the characteristic peaks of C-O-C in the cotton chemical structure. Compared to the spectrum of the original cotton, a new peak appears at 1730 cm^−1^ in the spectrum of the modified cotton that is attributed to the stretching vibration peak of the carbonyl group (C=O), indicating that methacryloxyethyltrimethyl ammonium chloride (METAC) has been successfully grafted onto the surface of the cotton.

**Figure 5:**
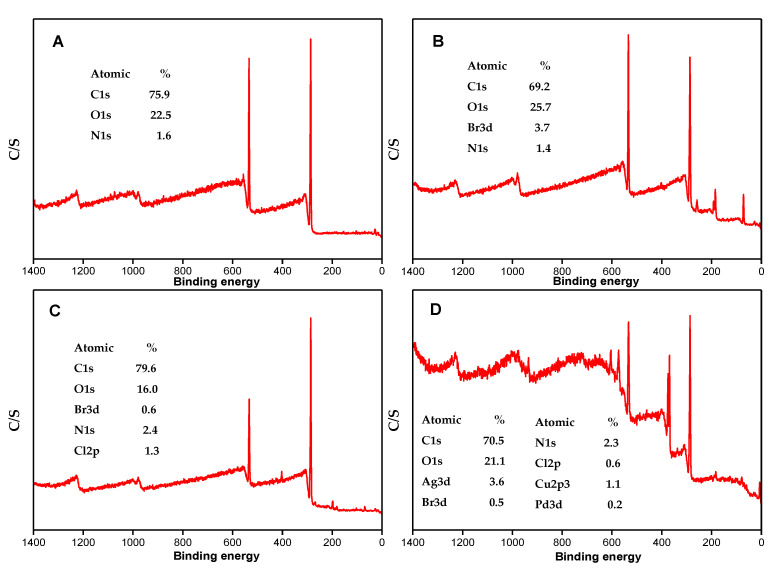
XPS of (**A**) pristine cotton, (**B**) Cotton-Br, (**C**) Cotton-METAC, and (**D**) Cotton-Ag.

The surface element chemical information investigation of the prepared cotton materials was performed by X-ray photoelectron spectroscopy (XPS). Figure 5 displays the XPS wide-scan spectra of the pristine cotton, Cotton-Br, Cotton-METAC and Cotton-Ag. The basic substrate of the cotton is cellulose, which consists mainly of C, H and O elements as indicated by XPS peaks for C 1s, N 1s, and O 1s at the binding energies of 285.9, 403.4, and 533.5 eV, respectively [29,30]. After bromination (Figure 5B), the Br 3d peak was observed at 69.5 eV, with the atomic percentage of 3.7%. The atomic percentage of C decreased from 75.9% to 69.2% due to the introduction of other elements into the cotton. Figure 5C shows the XPS results after METAC modification. A new peak at 198.9 eV that is related to Cl 2p is observed, indicating the successful immobilization of METAC on the brominated cotton [31]. The XPS result for the final product Cotton-Ag is shown in Figure 5D. The standard Ag 3d binding energy peak of silver is observed at 368.2 eV, with the atomic percentage of 3.60%. The XPS results verify the successful synthesis of the modified cotton via polymer brush grafting and the electroless deposition.

**Figure 6:**
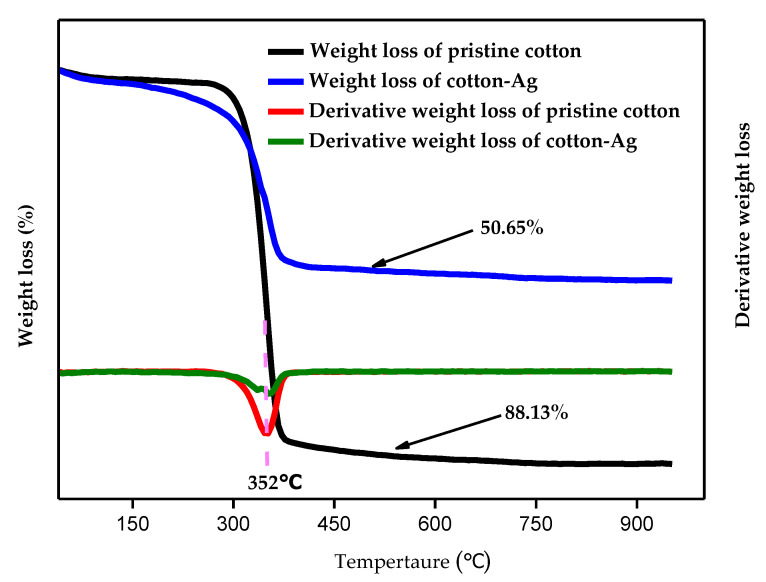
TGA analyses of the pristine cotton and Cotton-Ag.

**Figure 7:**
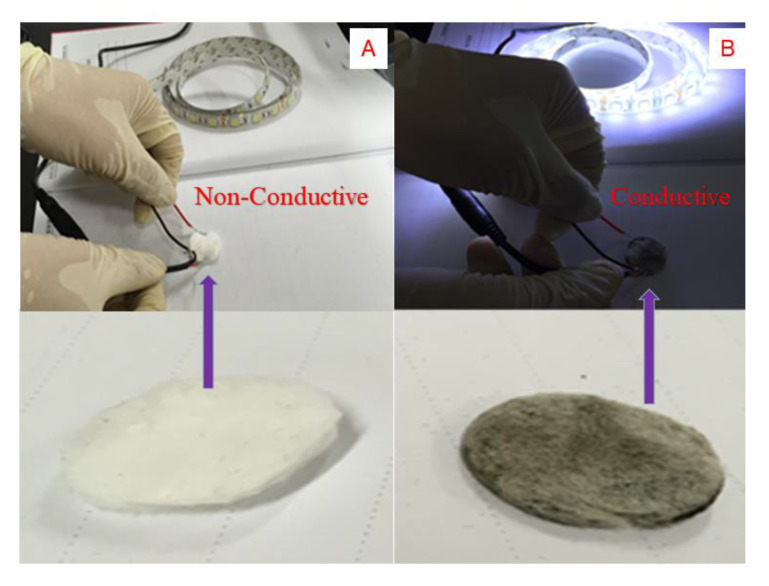
Conductivity test for (**A**) the pristine cotton and (**B**) Cotton-Ag.

The cotton before and after silver electroless deposition were examined by thermogravimetric analysis (TGA) with the results shown in Figure 6. The curves in Figure 6 are the TGA curves of the pristine cotton (black line) and Cotton-Ag (blue line). It can be concluded from Figure 6 that Cotton-Ag has a metallic residue (Ag-residue) which is the main origin of the low weight loss at high temperature. The derivative weight loss curves show an endothermic peak at around 352 degrees, and the Ag deposition on the cotton material does not significantly reduce the thermal stability of the neat cotton. Thus, the TGA results provide additional support for the successful modification of the pristine cotton in addition to the XPS and FTIR results described above. The deposition of Ag on the modified cotton (Cotton-Ag) can be further verified by the conductivity test, shown in Figure 7. Two conducting wires are connecting a power and an LED strip. When one end of one wire, and one end of the other wire, connect two sides of the prerpared cotton pellet along the direction of the diameter, it would form a closed circult. It can be seen that when the pristine cotton pellet was used to form the closed circult, the LED strip did not light up, indicating that the pristine cotton did not show conductivity. However, when Cotton-Ag pellet was used, the LED strip showed strong light, which was a clear evidence that the Cotton-Ag showed good conductivity due to the deposition of silver. 

**Figure 8:**
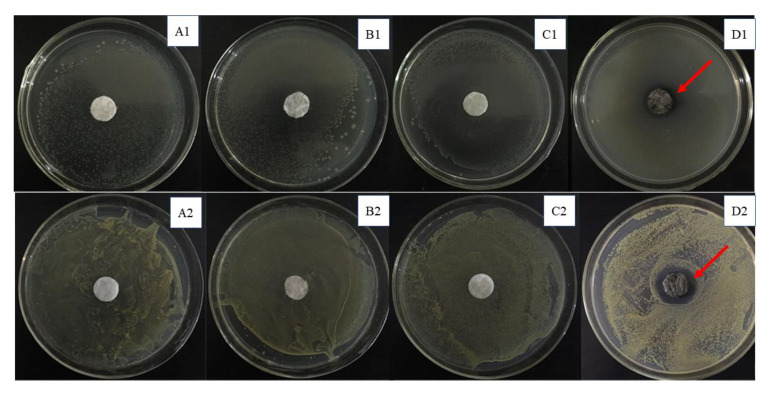
Inhibition of *Escherichia coli* (1) and *Staphylococcus aureus* (2) by the cotton at different stages of preparation: (**A**) pristine cotton, (**B**) Cotton-Br, (**C**) Cotton-METAC, and (**D**) Cotton-Ag.

In the culture dish covered with colonies, the inhibition zone with Cotton-Ag was clear as compared to that with other cotton-based materials, as shown in Figure 8. The diameters of the inhibition zone of *E. coli* and *S. aureus* can be easily measured with a vernier caliper, and the diameter of the zone was found to be greater than 16 mm. It was determined that the silver-deposited cotton in this experiment showed antibacterial activity toward the studied bacteria. Additionally, we found that the Cotton-METAC also had a slight antibacterial effect on *Escherichia coli*, but the diameter of the inhibition zone was less than 16 mm. The polymer on the Cotton-METAC is methacryloxyethyltrimethyl ammonium chloride, which is a quaternary ammonium salt. The structure of the quaternary ammonium salts has an N-site beside small aliphatic carbons that may act as a feed for bacteria. Therefore, these compounds show only weak negative effects toward bacteria [32].

**Figure 9:**
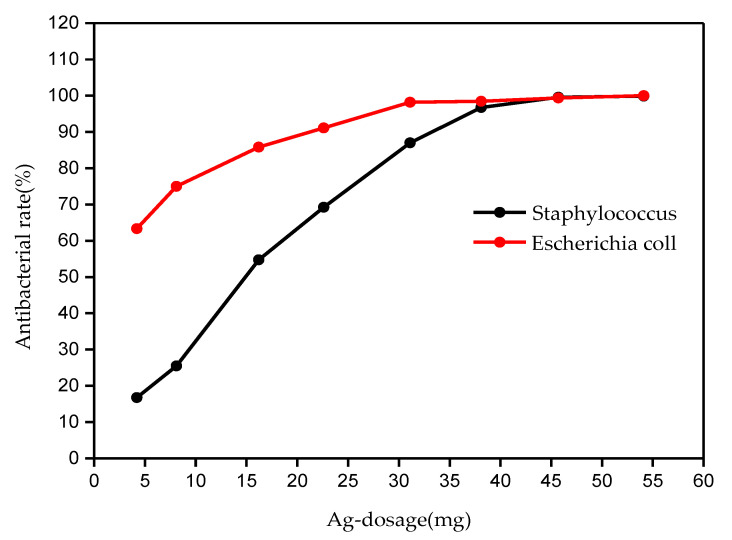
Relationship between the antibacterial rate and the Ag dosage on the silver-deposited cotton.

The inhibitory rate of the different silver dosages of the silver-deposited cotton for the studied pathogenic bacteria is shown in Figure 9. It can be concluded that the antibacterial rate increases with the increase of the dosage of the silver-deposited cotton. In addition, the silver-deposited cotton showed stronger inhibitory activity against Gram-negative bacteria (*E.* *coli*) than against Gram-positive bacteria (*Staphylococcus aureus*). This is because the Gram-positive bacteria have a thick cell wall (20–80 nm) and contain 40–90% peptidoglycan (15–50 layers). Most of these bacteria also contain a large amount of teichoic acid and protein, so their fat content is relatively small [33]. By contrast, the cell wall of Gram-negative bacteria is thin (10–15 nm) and contain only 1 or 2 layers of peptidoglycans. Its main component is the outer membrane, and it contains more protein. Therefore, the cell wall of the negative bacteria is more likely to be destroyed by the free silver ions from the silver-deposited cotton.

## 4. Conclusions

In this work, cotton was used as a substrate for the synthesis of a novel conductive and antibacterial agent. The Ag deposited cotton was prepared via the surface initiated-atom transfer radical polymerization (SI-ATRP) and the electroless deposition techniques. The novel modified cotton was studied using SEM, FTIR, TGA and XPS characterization methods. The optimal deposition time for electroless deposition of silver is 20 min. The designed cotton was found to be conductive in a closed circuit. Finally, the designed cotton was investigated as an antibacterial agent against Gram-negative and Gram-positive bacteria. The prepared Cotton-Ag exhibited excellent antibacterial performance against *E. coli* and *Staphylococcus aureus*. Quantitative experiments showed that the 40 mg dosage of silver on the modified cotton would achieve an antibacterial rate of 99% for both bacteria.

## Data Availability

The data presented in this study are available in article.

## Sample Availability

Samples of the materials are available from the authors.
